# Tracking the Track: The Impact of Different Grazing Strategies on Managing Equine Obesity

**DOI:** 10.3390/ani15060874

**Published:** 2025-03-19

**Authors:** Lorna Cameron, Meg Challinor, Sophie Armstrong, Abigale Kennedy, Sarah Hollister, Katharine Fletcher

**Affiliations:** 1 Equine Department, Hartpury University, Hartpury House, Gloucester GL19 3BE, UK; sophie.armstrong@hartpury.ac.uk (S.A.); abigale.kennedy@outlook.com (A.K.); 2 HorseWorld, Keynes Farm, Staunton Lane, Bristol BS14 0QL, UK; meg.challinor@horseworld.org.uk (M.C.); sarah.hollister@horseworld.org.uk (S.H.); 3 Welfare Aware, c/o Keynes Farm, Staunton Lane, Bristol BS14 0QL, UK; kate@welfareaware.com

**Keywords:** equine obesity, track grazing, equine bodyweight, equine behaviour

## Abstract

Horses evolved to live in herds, continuously moving and foraging. Domestic horses often have limited social contact and movement, with high calorific intake, contributing to obesity. “Track grazing” is an increasingly popular management system, but evidence of its impact on weight management, behaviour, and welfare is limited. Horses (*n* = 19) resident at HorseWorld (UK) were observed July to October 2023, monitoring bodyweight (kg), Body Conditions Score (BCS), hoof health, behaviour, and welfare indicators. Horses on track grazing systems lost significantly more bodyweight (kg) (*F*(1,15) = 8.752, *p* = 0.010) than their restricted grazing counterparts, although this was not supported by BCS findings which may be ineffective for subtle body weight changes. Horses on track grazing systems lost (mean ± S.D.) 10.67 ± 9.9 kg from their starting weight, whereas those on the traditional paddock systems gained 6.8 ± 13.7 kg. Hoof health and general welfare assessment scores were maintained. More behaviours in “positive”, “negative”, and “neutral” categories were observed in paddock system horses than those on the track grazing system, although these differences were not statistically significant. These findings suggest that horses prone to bodyweight gain can be managed effectively using track grazing systems whilst maintaining health and welfare.

## Simple Summary

Horses evolved to live in herds, moving and eating high fibre forage most of their time. Domestic horses often have limited movement, high calorie forage and social isolation. Track grazing systems, usually circular, fenced tracks around the perimeter of a paddock designed to increase movement and restrict grass intake, have increased in popularity. Promoted for management of horse bodyweight and other health issues, evidence of the impact of these systems is limited. This study observed horses in a charity setting at HorseWorld (UK), kept on either track systems or in traditional paddocks. Welfare indicators including hoof health, bodyweight, Body Condition Score (BCS), and behavioural measures were monitored. Horses kept on track grazing systems lost more bodyweight than their counterparts, although this was not supported by BCS findings, suggesting this may not be a sensitive enough measure. In general, there were no concerns on either grazing system regarding hoof health or general welfare and health assessments. There were no significant differences in behaviours observed between grazing systems. Track systems may be beneficial for horses requiring specialist weight management with limited effects on behaviour or welfare.

## 1. Introduction

Free-living horses evolved to forage on a range of plants, grazing for 16–18 h per day, consuming high volumes of fibrous forage [1], travelling as far as 16 km per day [2] in the company of a stable group of conspecifics [3]. Due to convenience and convention, traditional management practices often mean that horses spend an increased amount of time stabled. Pasture access is often modified grassland, where the sward has higher sugar content, with horses covering as little as 1.1 km per day [2] around a small paddock area. Stabling limits social contact between horses [4] and traditional pasture arrangements may increase the risk of aggression and injury. This limited contact with conspecifics may also be with inappropriately selected companions [5] at pasture. These factors have contributed to equine obesity rates, already an increasing health and welfare concern [6]. Traditional methods of controlling equine weight gain such as restricted grazing time, strip grazing, and grazing muzzles may also restrict natural social behaviours in the horse [7]. These restricted grazing methods may also increase the risk of dysfunctional social interactions and disordered nutritional intake [8]. Strip grazing and grazing muzzles are often perceived as having negative welfare outcomes and are prone to barriers to implementation [9]. Additionally, owners are often unable to recognise overweight horses [10,11] or are unequipped to make the effort to manage their horse’s weight effectively [12] whilst ensuring appropriate opportunities for social interactions with suitable equine companions [5].

Managing horses prone to weight gain can pose a range of challenges for the owner, including maintaining or reducing body condition. This can be further complicated when capacity to increase ridden exercise is not available, such as retired or injured horses [12]. Often the most commonly available and accessible method for horse owners to monitor body condition is via a recognised Body Condition Score (BCS) system [13,14] enabling them to identify when nutritional restrictions are required. For restricted nutritional intake to be maintained, whilst also allowing adequate social contact, appropriate movement and enrichment, the management challenges multiply even further [6].

New methods of grazing management may be warranted to maintain or reduce weight, but can be difficult to implement [15]. Sports and leisure horses are most often kept in relative social isolation [16]. This isolation can be further exacerbated when restrictions need to be placed on time spent at grass for weight management purposes. Anecdotally, the main beneficiaries of restricted grazing are horses with compromised metabolic systems, and with the increased prevalence of these issues, alternatives are warranted. Increased social isolation and limited movement can lead to a range of behavioural issues and welfare compromises for the horse [17]. These include, but are not limited to, stereotypy incidence [18], compensatory locomotor behaviours [19], unwanted handling behaviours [15,18], inter-individual aggression [20], and suppression of species-specific movement requirements [21]. However, these negative repercussions must be balanced against the health consequences that obesity can inflict on horses, such as impaired thermoregulation, exercise intolerance, laminitis [22], reduced fertility [23], and foal health [24].

In order to initiate weight loss in horses, nutritional intake should be reduced from 2–2.5% to 1.25–1.5% [6,25]. Even limited periods of turnout on good-quality grass can cause the horse to consume more forage than those consistently on little grass [7,26], so may be counterproductive. Many traditional restricted grazing practices, such as strip grazing, can also compromise the horse’s basic needs for forage, friends, and freedom, and may pose a serious welfare challenge [27].

The track grazing system is one approach that may balance competing priorities and reduce the prevalence of equine obesity. Track grazing systems are usually circular, fenced tracks around the perimeter of a paddock designed to increase movement and restrict grass intake [15]. Limited research has been undertaken in the effectiveness of track grazing systems in equine weight management and what impact, if any, they may have on equine behaviour at pasture. An increasing interest in and usage of track grazing systems has been reported by survey respondents [9,15] but information on best practice guidelines and efficacy of these systems for equine weight management is limited at present. Recent research has identified that horses kept on a track system moved more than those in restricted grazing management systems and showed less overtly aggressive behaviours [28]; however, in the relatively short exposure time to each grazing system, no significant differences in body weight or Body Condition Score (BCS) were observed.

Researching the impact of differing grazing strategies on obesity management in vulnerable horses can be challenging as any change to nutritional intake may adversely impact weight gain or loss [29] and as adiposity is often driven by genetic factors, any change can be even more difficult to reverse. Other confounding factors such as the health of the gastrointestinal microbiome [30] can be influenced by pasture changes, further impacting weight management. Yet, to drive appropriate management choices for owners or carers of horses that tend towards obesity, whilst still meeting their needs for social interaction, forage, and freedom, further evidence is required [31] over a longer experimental period.

To this end, this study aimed to utilise equine management data normally collected from the HorseWorld (UK) Charity (Bristol, UK) population and observe equine behaviour on different grazing systems to add to current knowledge of track system grazing, whilst avoiding any unnecessary impact on the existing management of the population of rescued horses and ponies. It was hypothesised that horses grazing on track systems would reduce in bodyweight over the summer period whilst maintaining welfare and health levels.

## 2. Materials and Methods

Following institutional ethical approval (Hartpury University Ethics Committee, ETHICS2022-153), horses were assigned to the observational study from the existing population of rescued or retired horses from HorseWorld (UK) by appropriate staff responsible for their management, health, and welfare.

### 2.1. Sample

Nineteen horses of mixed breeds and types were allocated to the project (geldings n = 6, mares n = 13), aged (mean ± S.D.) 13.6 ± 6.3 years, height 122.1 ± 22.4 cm with various weight management requirements based on existing health conditions (Table 1). These horses, assessed as susceptible to excessive weight gain by the attending veterinary surgeon and HorseWorld (UK) staff, or with health conditions that preclude increased exercise to aid in weight management, were placed on one of three track grazing systems or a small range of traditional restricted grazing paddocks at the beginning of the summer grazing routine as per the HorseWorld (UK) standard grazing regime (Supplemental File S1). Any horses involved in ongoing legal proceedings during the observation period or requiring alternative management due to veterinary concerns were excluded from the study population. Any horses displaying a deterioration in health or a failure to thrive, as assessed by routine HorseWorld (UK) assessments, in their respective grazing system were removed from the study as is normal procedure at this charity.

### 2.2. Weight and Welfare Tracking

All horses allocated to the project were initially weighed on one of two equine weighbridges (Olympic model, Horse Weigh, U.K.) and body weights were recorded, then weighed subsequently every month during the duration of the project as per standard HorseWorld (UK) monitoring procedures to enable a weight range to be assigned to each horse to take seasonal variation into account. This monthly monitoring also included Body Condition Scoring (BCS) [14] by two observers from the HorseWorld (UK) staff trained by the veterinary surgeon. A standardised HorseWorld (UK) Welfare Assessment was completed for each horse monthly, identifying any health or welfare issues by a trained staff member to identify any horses that required removal from the study. Thirteen horses were allocated to track system grazing (geldings n = 2, mares n = 11) aged (mean ± S.D.) 13 ± 6.94 years, height 116.68 ± 18.78 cm, weight 316.69 ± 115.91 kg, BCS 3.71 ± 0.4. Six horses were allocated to restricted paddock grazing (geldings n = 4, mares n = 2) aged (mean ± S.D.) 14.83 ± 5.04 years, height 133.77 ± 26.82 cm, weight 452.17 ± 188.74 kg, BCS 3.8 ± 0.57. All horses were allocated to grazing systems by HorseWorld (UK) staff according to their established assessment of the social, health, and welfare needs of each individual horse within their care.

### 2.3. Hoof Health Tracking

At each farriery appointment, foot health, condition, and growth were assessed by a qualified farrier familiar with the horses to produce a hoof health score at intervals throughout the study. Comments on general hoof quality for each foot were scored from 0 to 5 (0 as worst hoof health, 5 as optimal hoof health). Further detailed assessment included growth, scored 1 for optimal and 0 for not optimal; presence of issues, scored 0 for presence of chips, splits, or cracks and 1 for absence of these; thrush, present as 0 and absent as 1; bruising, scored 0 for present and 1 for absent; and overall farrier comments, scored 1 for positive, 0 for no comment, and −1 for a negative comment. This gave a possible “healthy hoof” score of 10 for each hoof, thus adding up to a maximum hoof health score of 40 per horse for each assessment.

### 2.4. Behavioural Tracking

Horses were observed twice per week from July 2023 to October 2023, resulting in 162 behaviour observations (37.5 h), when the track systems were being utilised within the day-to-day management of the population of horses at HorseWorld (UK). The horse groups on each grazing system were observed for 10 min, recording general behaviour and any social interactions utilising an ethogram specifically developed by the HorseWorld (UK) team for the project (Table 2). The ethogram was developed from the published literature for ease of use by HorseWorld (UK) staff. A small team of trained observers used continuous focal sampling of each individual to record observed behaviours from the same position outside of the track or paddock to avoid undue impact on the behaviours of the horses and at as similar a time of day as possible within their daily routine. Behaviours observed were logged utilising a “one zero” method indicating presence or absence during the observation periods. Data were transferred from the recording sheets to an Excel Spreadsheet and descriptive analysis allowed a behavioural profile to be developed for each group of horses. These were then averaged for the group to account for the differences in group sizes.

Behaviours were categorised into “positive”, “negative”, and “neutral” behaviours to allow for further behavioural analysis (Table 2). Behaviours were categorised as “positive” if they were likely to reduce the risk of injury, increase social cohesion, or be indicative of positive affective state. Likewise, behaviours were categorised as “negative” if they were likely to increase the risk of injury, reduce social cohesion, or be indicative of negative affective states. Finally, behaviours were categorised as “neutral” if the evidence available at the time of the study was contradictory or unclear.

### 2.5. Position Tracking

**Figure 1:**
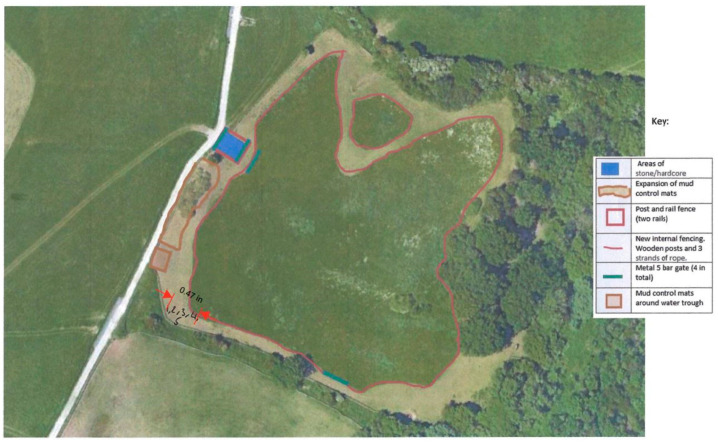
Example map of Track A attached to behavioural observation sheets with study horse positions approximated.

Horses were assigned to grazing systems by the HorseWorld (UK) staff based on their requirements (Table 1) consisting of either a traditional restricted grazing system or a track grazing system (Table 3). During each behavioural observation, observers recorded the position of each subject in the paddock or track system on a standardised printed Google Maps™ image of the particular area at the start and end of the observation period (Figure 1).

Marked maps were then scanned to a Portable Document Format (PDF) and, using the measuring tool in Adobe Acrobat, the distance between participant horses was measured and converted to metric measures. As horse positions were marked on most, but not all, assessment sheets at the start and end of each observation, the greatest spread of sample horses was used in each case.

### 2.6. Data Analysis

Monthly assessments of body weight, BCS, welfare status, and foot health were collated for further analysis and any anomalies noted. There were no welfare concerns raised within the regular welfare assessments for any other horses during the course of the study. All horses within the project were weighed on an equine weighbridge at the start of data collection in early July 2023 and subsequently in early August, late August, and late September 2023 to align with normal monitoring procedures at HorseWorld (UK), resulting in four weight assessments.

All statistical analysis was performed using Statistical Package for Social Sciences SPSS version 19. Data were tested for normality using the Shapiro–Wilks test with only body weight and body weight change meeting the assumptions for parametric testing (*p* > 0.05). Differences in equine bodyweight at the start of the project versus the end of the project were tested using a one-way ANOVA and t-tests for independent samples with an α level of *p* < 0.05. Behaviour data, farrier assessment, and position tracking were tested for differences utilising Friedman’s ANOVA with post hoc analysis utilising a Mann–Whitney U for behavioural data and position tracking. Post hoc tests for repeated farrier assessments used Wilcoxon tests all at the same α level of (*p* > 0.05).

## 3. Results

One horse was removed from the study due to health concerns not associated with the programme of research, and data for the remaining subjects (*n* = 18) were collated for further analysis.

### 3.1. Weight and Welfare Tracking Analysis

#### 3.1.1. Body Weight and Body Condition Score (BCS) Tracking

**Figure 2:**
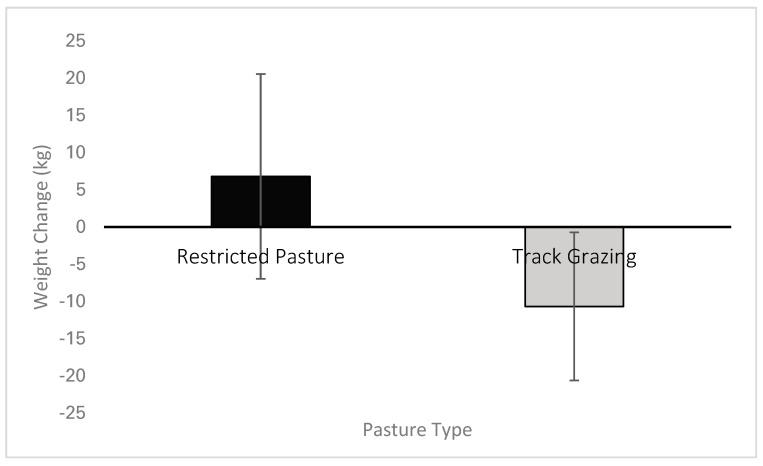
Mean bodyweight change of restricted pasture versus track grazing groups.

When comparing each horse’s July bodyweight versus late-September bodyweight there was a statistically significant difference between grazing management groups determined by one-way ANOVA (*F*(1,15) = 8.752, *p* = 0.010). Horses on track grazing systems lost (mean ± S.D.) 10.67 ± 9.9 kg of bodyweight, a significant loss from their starting weight (*t*(11) = −3.714, *p* = 0.02), whereas those on the traditional paddock systems gained 6.8 ± 13.7 kg, although this was not a significant gain (*t*(4) = 1.105, *p* = 0.331) (Figure 2). Even when adjusted for initial bodyweight, those horses kept on the track system lost an average of 3.4% of their initial bodyweight compared to those on the traditional paddock grazing system who gained an average of 1.5% of their bodyweight over the period of data collection.

**Figure 3:**
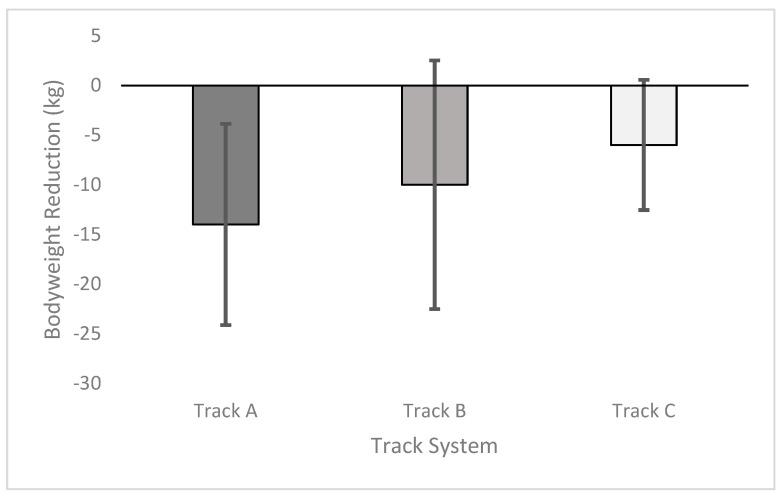
Bodyweight reduction by track system.

The greatest bodyweight (kg) loss (mean ± S.D.) was recorded in the horses on Track A (14 ± 10.12), followed by Track C (10 ± 12.52), then Track B (6 ± 6.56) (Figure 3), although not statistically significant.

Body Condition Scores (BCSs) reduced by 0.5 in 25% of horses on track grazing and increased by 0.5 in 40% of the horses on paddock grazing, with all other BCSs staying constant throughout the data collections. There were no statistically significant differences in BCS results for either the track system group (χ^2^(3) = 3.33, *p* = 0.343) or the paddock grazing group (χ^2^(3) = 3, *p* = 0.392).

#### 3.1.2. Farriery Assessment

Farrier assessments were completed throughout the data collection period as and when farrier attention was due to the horses within their normal management routine. Sixty eight percent of track system horses (*n* = 4) with repeated farrier assessments increased hoof health score and the remainder maintained their score. Initial mean hoof health score for track system horses was 33.17 (fore hooves = 16.17, hind hooves = 17) and final mean hoof health score was 34.67 (fore hooves = 17, hind hooves = 17.67). Horses kept on paddock grazing with repeated farrier assessments available (*n* = 2) either maintained a consistent farrier score throughout (n = 1) or reduced hoof health score marginally; initial mean hoof health score for paddock horses was 30.5 (fore hooves = 15, hind hooves = 15.5) and final mean hoof health score was 29.5 (fore hooves = 14, hind hooves = 15.5).

### 3.2. Behavioural and Position Tracking Analysis

Behaviour observations and position tracking were assessed between grazing condition systems.

#### 3.2.1. Behavioural Assessment

**Figure 4:**
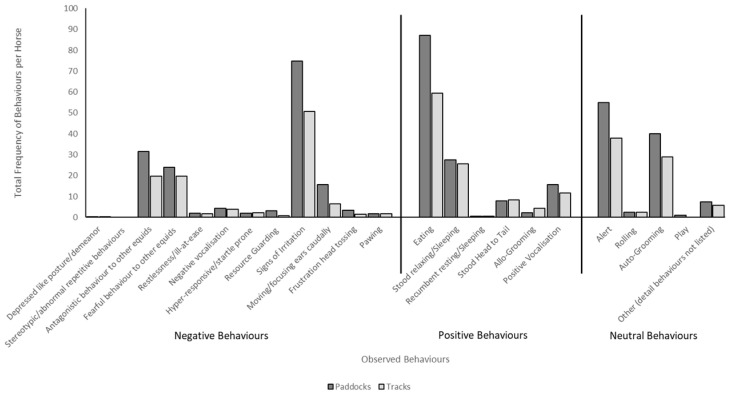
Frequency of behaviours per horse observed on the track and paddock systems.

Behaviours observed per horse were collated (Figure 4). All behaviours were observed at least once with the exception of stereotypical behaviour, with none observed by any horses in the study period.

**Figure 5:**
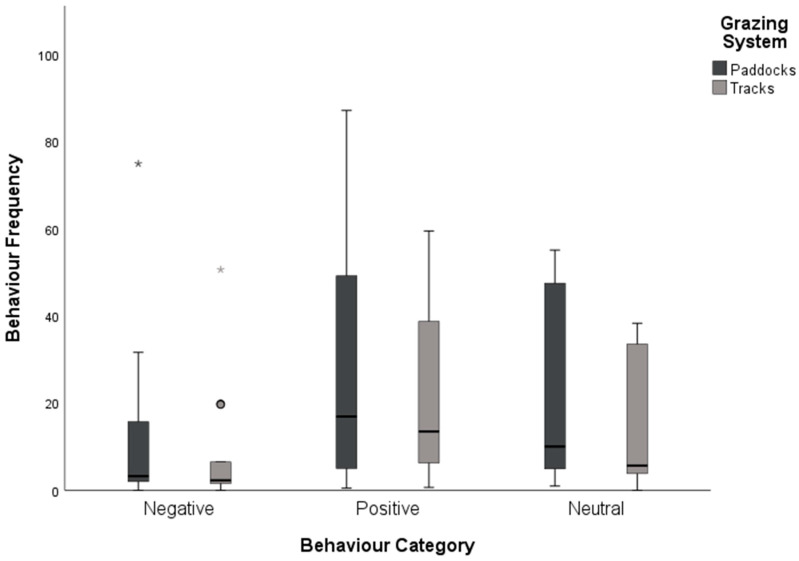
Box and whisker plot of median values for negative, positive, and neutral behaviours per horse on track grazing and paddock grazing systems (o denotes mild outliers, * denotes extreme outliers).

Behaviours were totalled for track systems and traditional paddock systems and further categorised as “positive”, “negative”, or “neutral” behaviours (Table 3). Total behaviours observed were compared between the track grazing systems and the traditional paddock grazing systems. As the groups of horses were of unequal size, each count of behaviours observed was divided by the number of horses in the sample, giving a behaviour count per horse for each behaviour (Figure 5). Although not significantly different, behaviours per horse were impacted by grazing system with those on the track system demonstrating fewer observed behaviours overall.

#### 3.2.2. Position Assessment

**Figure 6:**
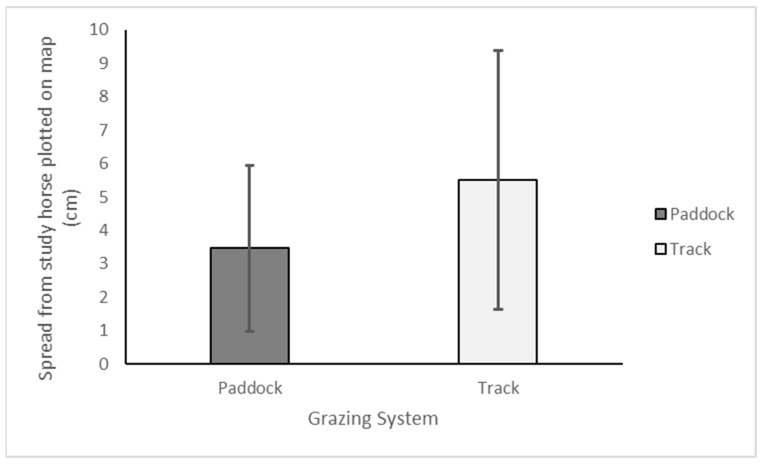
Spread from study horse plotted on map (cm).

Horses were located over a larger distance between horses on the track system grazing compared to the paddock system grazing; however, this difference was not significant (Figure 6).

## 4. Discussion

This study found clear evidence that horses susceptible to gaining bodyweight in the summer period when the sward is at its most nutritious and calorific can be managed effectively utilising track grazing systems, reducing bodyweight during a summer season whilst maintaining hoof health and general welfare, and limited impact on what is considered the expected behavioural repertoire of horses kept at pasture.

### 4.1. Management of Equine Bodyweight

Horses kept on the track systems reduced bodyweight more than those kept on a traditional paddock system. Maintaining or reducing bodyweight in horses susceptible to weight gain, perhaps with associated metabolic challenges such as Equine Metabolic Syndrome (EMS) or a propensity for laminitis [7], can prove difficult and traditional methods such as restricted grazing or the use of grazing muzzles [8] can inhibit normal equine social interactions between the herd. The management of these horses is an ongoing challenge for many horse owners, and accessible and practical information is, at times, sparse. This study has shown that the introduction of a track grazing system may prove effective in helping equine weight management, particularly when increased exercise is not appropriate for the individual due to age or injury. It should be noted, however, that horses within the present study were those that were most in need of effective management of their bodyweight, but as bodyweight gain has been linked to a reduction of insulin sensitivity in horses [44], rapid weight loss may be indicated to avoid insulin dysregulation (ID). The increased weight loss seen in this population of horses kept on a track grazing system versus traditional paddock management suggests that the adoption of track grazing systems may prove useful in the management of horses prone to weight gain or with complex metabolic issues, whilst still allowing them access to the three “Fs” first suggested by Lauren Fraser: friends, forage, and freedom [45]. Although horses on track grazing lost significantly more body weight than those on paddock grazing, it is unclear if this weight loss was biologically significant to these individuals leading to any health benefits. It should be noted that all track systems did not perform equally in reducing equine bodyweight. The track system that induced the greatest weight loss within the sample population was Track A, which has the most undulating ground with a steep incline on two sides and resources available at different altitudes, i.e., natural water and shade at the bottom of the slope and access/extra forage resources at the top. This may well have encouraged greater mobility of the horses, but access to tracking this movement was not available. Indeed, Kirton et al. [28] found that ponies fitted with Global Positioning System (GPS) trackers moved for significantly more time and greater distances when on a track system versus a strip grazing system in a repeated measure, cross-over design. It is suggested from the findings of the current study that increased movement may well be key to effective weight loss, but future research with the HorseWorld (UK) equine population is warranted utilising validated GPS trackers to establish why Track A was the most effective in reducing equine bodyweight over the summer period.

The differences seen in weight loss between grazing systems could also be explained by other confounding factors beyond the grazing system arrangement. The grazing density within track systems was greater due to larger groups of horses, a factor which may have impacted time spent grazing, available digestible energy (DE) [21], and behavioural interactions. These factors should be considered in future studies to establish if it is the track design that is impactful rather than grazing density and group size.

Although the horses selected for grazing on the track system may well have been, inadvertently, those that required the greatest reduction in bodyweight to maintain optimal health, when data were adjusted to a percentage of initial bodyweight, those on the track system still lost more weight during the data collection period. It should be noted that the monitoring of Body Condition Score (BCS) in these horses, even although this was undertaken by trained observers, did not reflect bodyweight loss by the horses during this time period. This would suggest that monitoring equine bodyweight via BCS methods, even by trained observers, is not a reliable method to monitor more gradual changes in equine weight, particularly in horses susceptible to weight gain. In future research, the more sensitive 1–9 scoring system may be appropriate [13] as opposed to the 1–5 scale [14] utilised in this study, which may not have been sensitive enough to clearly track the subtle changes of equine bodyweight recorded over a period of just a few months. This would suggest that further research is warranted to help inform and develop more effective methods to monitor equine bodyweight. The everyday horse owner may not have access to a weighbridge on a regular basis and as such may rely on BCS to monitor equine weight gain or loss. There were no significant changes in hoof health reported, with all but one horse either maintaining or improving hoof health regardless of grazing system. A marginally greater improvement in hoof health in those horses managed on the track grazing systems was observed. This small improvement in hoof health may well be related to increased movement on the track grazing systems, but further research is needed utilising GPS tracking of movement duration and distance on both restricted and track grazing systems in future.

Bodyweight changes observed via weighbridge measures were not reflected in BCS, even when assessed by experienced and trained observers. This suggests that further research is warranted to investigate the efficacy of BCS systems in tracking bodyweight change in horses susceptible to weight gain, having metabolic issues, or prone to laminitis to better inform horse owners in accessible methods of monitoring the condition of their horses.

### 4.2. Impact on Horse Equine Behaviour and Position

A common concern when introducing new or innovative equine management systems is the possible impact on equine behaviour and safety. During this study, there were no significant differences in equine behaviour displayed between grazing systems, although these were independent groups, and future research should consider a cross-over design; however, this was not considered appropriate in this particular study sample. When the behaviours were grouped together into “positive”, “negative”, and “neutral” behaviours, all categories reduced with the horses kept on the track grazing systems. Categorisation of these behaviours is open to debate, however, as some behaviours may be context-specific, moving categories depending on prior and post behavioural expression, e.g., “play” was categorised as a “negative” behaviour as this behaviour is seldom seen in feral situations between adult horses. McDonnell and Poulin [40], however, suggest that play behaviours may be important in the behavioural repertoire of domestic horses. It should be noted that the majority of horses kept at pasture in the United Kingdom (UK) are kept in comparatively small groups [5] which may be better represented by the horses kept in the restricted grazing groups. The limited nature of the behavioural observations in this study are likely not representative of the behavioural repertoire over a 24 h period [28]. Individual horses could have impacted the behavioural observations and, as behaviour was reported as a group average to account for group size, individual horses could have adversely impacted these results, as could the natural rate of behavioural occurrences. The ethogram was not fully validated prior to its use, although a pilot study utilising video analysis for training purposes was utilised and this may well have impacted the accuracy of some of the measures used. Validation of the ethogram and the limited nature of behavioural observations were limited by the availability of staff with more responsibilities to the welfare of the horses than research observations. GPS trackers and activity trackers may provide a good proxy for the costly time spent in observation, although Bartlett et al. [5] highlighted that real-time observations were the gold standard, even if expensive in staff time and effort.

The distance between horses was greater for those in the track grazing systems, possibly suggesting that the increased weight loss seen in this cohort may be due to increased activity encouraged by the grazing system design and spread of valuable resources, as seen by Kirton et al. [28]. This would suggest that further research is warranted within a more controlled environment to observe the same group of horses both on a traditional paddock system and on a track grazing system, whilst maintaining good welfare for all research subjects. This was not considered appropriate within this particular cohort of rescue horses, but future research would be advisable in a less vulnerable population of horses. There was also limited consideration of more detailed welfare assessments such as the Five Domains Model [46], and this is advised in future studies; however, these assessments had to be easy to apply for busy staff with competing priorities. Utilising technology such as infra-red cameras, particularly around valued resources such as forage, water, or enrichment, may add depth and detail to future studies, then be applied to the management of vulnerable equine populations such as these.

Researching within the confines and requirements of an operational equine welfare charity proved not only challenging, but also extremely rewarding. It allowed the research team to gather “real-world” data applicable to both those either working in the equine rescue charity sector and to horse owners facing the challenges of managing horses that are susceptible to weight gain. Most horse owners want to manage their horses well, but report facing barriers in terms of published research that “works” in the real world and from “resistance” from established practitioners and practices [16].

### 4.3. Further Steps

Future research should focus on the impact of grazing system on equine bodyweight, welfare, activity levels, and behaviour. More information is needed by horse owners to better inform their management choices to further support their efforts to improve the lived experience of their horses whilst still managing individual health needs. The way we manage horses needs to be improved in future to allow us to better ensure their welfare, considering both their physiological and psychological needs to promote not just an acceptable life, but a good life that is healthy and safe for all.

## 5. Conclusions

Research in real-world situations can prove challenging, yet the data generated are practically applicable and can have benefits for the equine industry. Horses susceptible to bodyweight gain or with metabolic issues lost significantly more bodyweight in the track grazing systems than similar horses kept on traditional restricted grazing systems, whilst not significantly impacting their behaviour, social interactions, hoof health, or general welfare. These findings suggest that track grazing systems will prove useful in effective equine bodyweight management in vulnerable horses and future research should utilise technological advances to establish which aspects of equine activity on track systems influence bodyweight the most.

## Figures and Tables

**Table 1:** Sample population overview.

Field	Horse	Sex	Breed	Age (Years)	Height (cm)	Known Health Conditions/Management
Track A	1	Mare	Cob	11	143.26	Prone to weight gain
	2	Gelding	Cob	9	121.92	Prone to weight gain
	3	Mare	Welsh Sec A	16	111.76	Prone to weight gain
	4	Mare	Cob	4	113.79	Prone to weight gain
	5	Mare	Cob	3	123.95	Prone to weight gain
	6	Mare	Welsh Sec A	13	114.8	Prone to weight gain
Track B	7	Mare	Shetland	7	81.28	None
	8	Mare	Shetland	9	91.44	Partially collapsed trachea - limited exercise options for weight management
	9	Mare	Shetland	14	91.44	Laminitis and pituitary pars intermedia dysfunction
Track C	10	Mare	New Forest x	26	134.11	Laminitis, pituitary pars intermedia dysfunction and asthma
	11	Mare	TB x Trotter	22	134.11	Laminitis
	12	Gelding	Welsh Sec B	14	122.94	Laminitis and prone to weight loss
	13	Mare	Welsh x	21	132.08	Equine metabolic syndrome
Paddock D	14	Gelding	Cob x Trotter	8	152.4	Prone to weight gain
	15	Gelding	Cob x	15	162.56	Navicular and asthma
Paddock E	16	Gelding	Shetland	11	91.44	Prone to weight gain
	17	Gelding	Shetland x Cob	20	111.76	Prone to weight gain
Paddock F	18	Mare	New Forest x	21	142.24	Laminitis, breathing issues
	19	Mare	Cob x	14 (estimated)	142.24	Laminitis and prone to weight loss

**Table 2:** Ethogram developed by HorseWorld (UK) staff used for behavioural observations.

Behaviour	Description	Behaviour Category
Depressed like posture/demeanour	Less responsive to the environment than what is normal for that horse. Often with a ‘zoned out’, worried, or glassy-eyed staring facial expression [32].	Negative
Stereotypies/Abnormal Repetitive Behaviours	Including abbreviated weaving, nose tossing/flipping, head bobbing, frequent yawning bouts, sympathetic surge resolution signs, crib biting, wind sucking, and lip smacking [32,33].	Negative
Antagonistic Behaviour to other Equids	Behaviour observed including—Bite threat, biting, bump/pushing, rearing, chasing, ears pinned, head threat, kick threat, kicking, or herding [34]. Descriptions of each: - Threat—consisting of lateral pinned back ears, arched neck, and/or movement of the head/rear towards the opposing horse, but with no physical contact [35]. - Bump or Push—making forceful contact with another horse using the head, neck, or shoulder [35]. - Chasing—horse chasing another at speed to displace the other horse from the immediate area or with the intent of engaging the animal in more agnostic behaviours [35]. - Biting/Kicking—involves physical contact [35]. - Rearing—lifting of forelegs off the ground as the body is elevated into a more vertical position. Rearing in itself is a threat with the potential for further agnostic behaviours such as striking and boxing behaviours [35].	Negative
Fearful Behaviour to other Equids	Exhibited by the loser of the antagonistic encounter. May be expressed by running away, laid down ears, lowered head posture, lowering of the hind quarters, and sometimes jaw snapping (however often only observed in juveniles). Submissive behaviour [35].	Negative
Restlessness/Ill-at-ease	Including changing activities frequently (foraging, standing rest, standing alert) more often than would be expected, circling/pacing, fidgeting, frequent repositioning during recumbency, or abandoning recumbency/elimination attempt [32].	Negative
Negative Vocalisation	Including groaning, sighing, grunting, screaming/calling, and teeth grinding. Squeals have been noted to be produced in agonistic reactions [32,36].	Negative
Hyper-Responsive/Startle Prone	Lower threshold and more animated reaction to environmental stimuli [32].	Negative
Signs of irritation	Often seen due to insects. Swishing/flicking tail—moving the tail suddenly from side to side [32]. Swatting/batting—swinging the head and neck at a particular area of the body [32].	Negative
Moving/Focusing Ears Caudally	Moving the ears to focus backwards or laying the ears back against the neck [32].	Negative
Frustration Head Tossing	Quick rotational toss of the head, similar to a head threat [32].	Negative
Pawing	Front leg is lifted, then extended quickly in a forward direction, followed by a movement backward dragging the toe against the ground in a digging motion [37].	Negative
Eating	Grazing—Ingest grassy vegetation. With the lips and tongue, vegetation is gathered into the mouth, broken off usually in clumps by jerking the jaw while chewing, and swallowed [38]. Browse—Ingest woody plants [38].	Positive
Stood Relaxing/Sleeping	- Sleeping—with eyes closed, and head lowered below the back, light sleep in a standing position, often bearing weight on three legs (one leg slightly flexed) [38]. - Resting—standing inactive in a relaxed posture with head slightly lowered, eyes partly or nearly closed, and often bearing weight on three legs (one hind leg slightly flexed). With deeper drowsiness, the lips relax, and ears rotate laterally. If recumbent, then in a relaxed position and aware of surroundings but not in a stage of sleep [38].	Positive
Recumbent Relaxing/Sleeping	- Sleeping—horse is lay down and in the stages of sleep. Less aware of environment [38]. - Resting—horse is lay down but still aware of surroundings and not in stage of sleep [38].	Positive
Alert	Standing still with head high, ears pointing forward. Nostrils may or may not be dilated [39].	Neutral
Allo-Grooming	Lateral parallel body position of two horses that allows for nibbling along the back or withers of each horse [35].	Positive
Auto-Grooming	Nibbling, nuzzling, and/or biting at an area of the body, or rubbing one part of the body to another or against an object [32].	Neutral
Play	Consists of several behaviours categorised by object play, sexual behaviour, locomotor play, and play fighting [40]. - Object play—involves contact and manipulation of an object, and can be an inanimate object such as something in the environment, or animate such as mane, tail, or body part of another animal [40]. - Play sexual behaviour—usually common in foals and young adolescents of both genders. Most common play behaviours include elimination marking sequence, teasing, and mounting [40]. - Locomotor play—any behaviour of play performed in motion, including frolicking, running, chasing (with no agnostic intentions), bucking, jumping, and prancing [40]. - Play fighting—similar to adult fighting behaviour but with more of a sporting character. Observed to alternate defensive and offensive roles unlike serious fighting. Consists of nipping/biting anywhere on the body, wrestling, pushing, rearing, and evasive spins [40].	Neutral
Stood Head to Tail	Can also be characterised as huddling. Provides physical protection as well as being a resting behaviour and allowing for insect control around the head by tail swishing effect of neighbours [35].	Positive
Rolling	Laying down to sternal recumbency, rotating from sternal to lateral, onto dorsal recumbency [32].	Neutral
Positive Vocalisation	Snorts and shorter, lower frequency whinny. And nickers [36,41,42].	Positive
Resource Guarding	Management conditions where available resources such as food are restricted, small enclosure sizes, and/or high density of horses and unstable group membership are usually associated with higher aggression levels, and guarding of available resources [43].	Negative
Other	Any behaviour that has not been listed previously.	Neutral

**Table 3:** Table of available grazing areas in each assigned area.

Grazing Area Information				
Field	Perimeter (m)	Total Area (Acres)	Area Available for Grazing (Acres)	Incline
Track A	551.87	4	1.82	Very steep on sides
Track B	476.89	3	0.77	Very slight
Track C	383.92	2	0.45	Flat
Paddock D	594.68	4	3.98	Flat
Paddock E	708.97	8	7.24	Flat
Paddock F	414.63	2	1.99	Flat

## Data Availability

The data presented in this study are available upon request from the corresponding author.

## Supplementary Materials

The following supporting information can be downloaded at: https://www.mdpi.com/article/10.3390/ani15060874/s1, Figure S1: Track A Area, Figure S2: Track B Area, Figure S3: Track C Area, Figure S4: Paddock D Area, Figure S5: Paddock E Area, Figure S6: Paddock F Area, Figure S7: Track A Layout, Figure S8: Track B Layout, Figure S9: Track C Layout.
