# Supplementary material for: Tracking the Track: The Impact of Different Grazing Strategies on Managing Equine Obesity
Source: Animals (Basel). 2025 Mar 19;15(6):874. doi: 10.3390/ani15060874 (PMC11939410; doi:10.3390/ani15060874)

Figure S1 - Track A Area

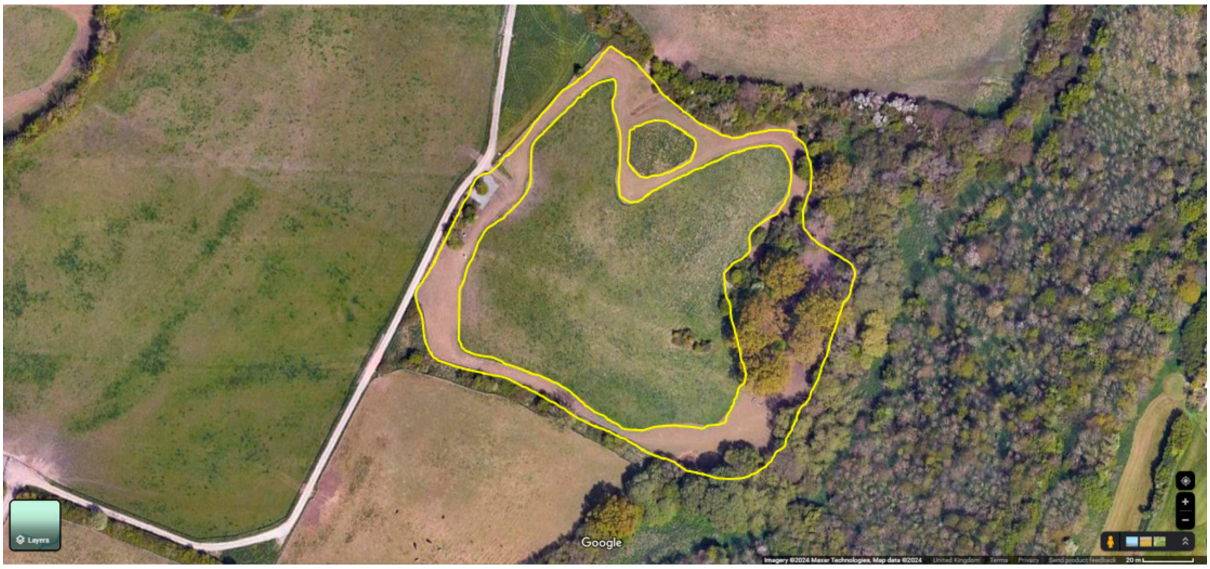

Figure S2 - Track B Area

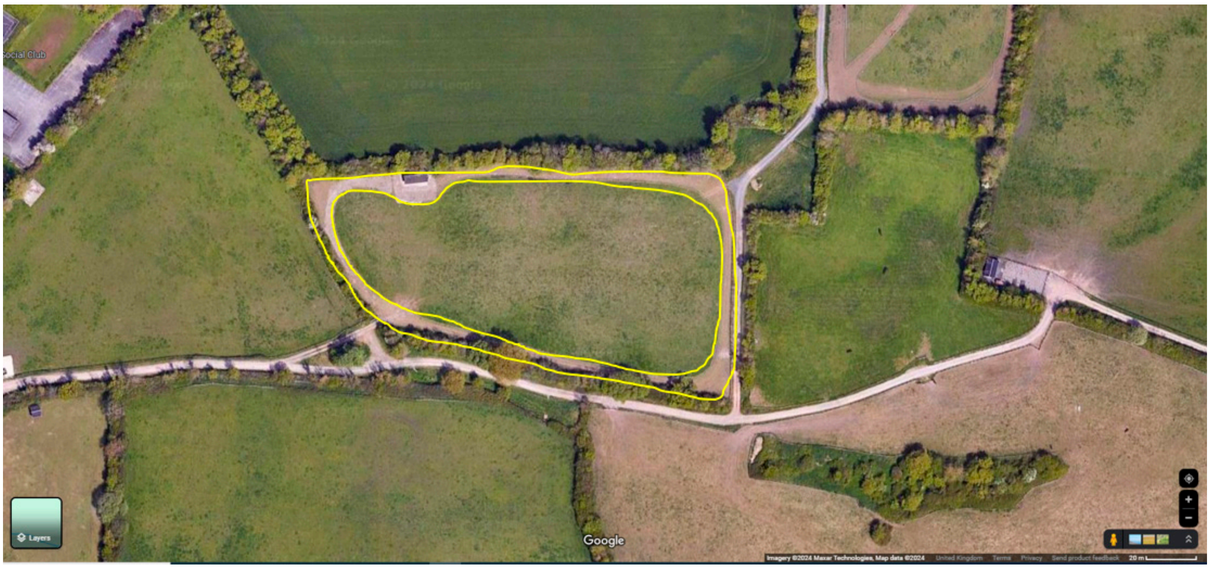

Figure S3 - Track C Area

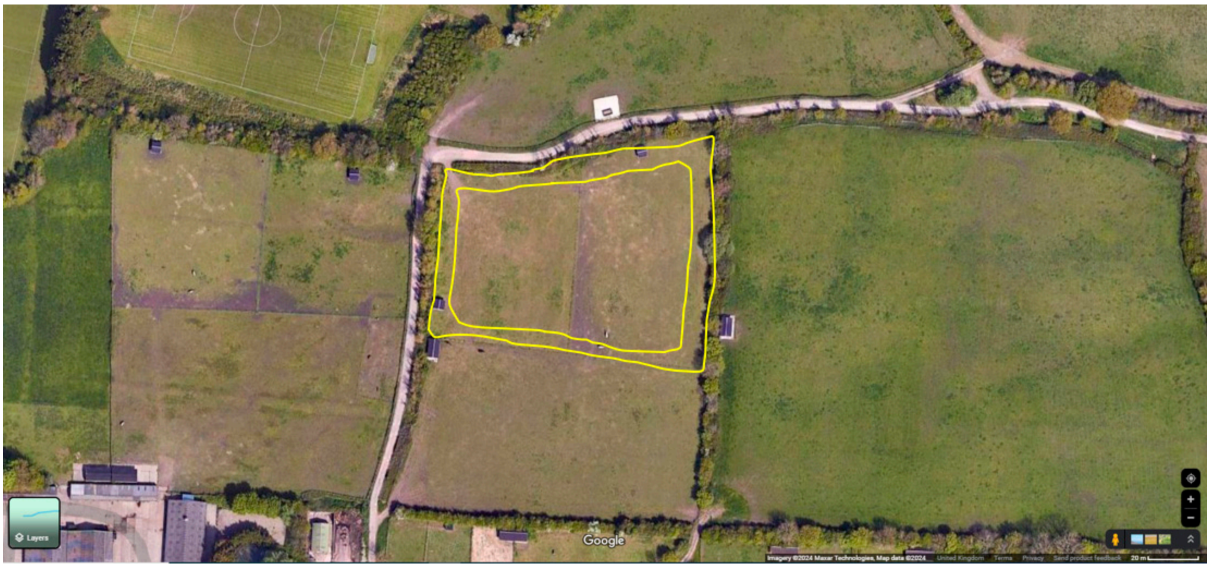

Figure S4 - Paddock D Area

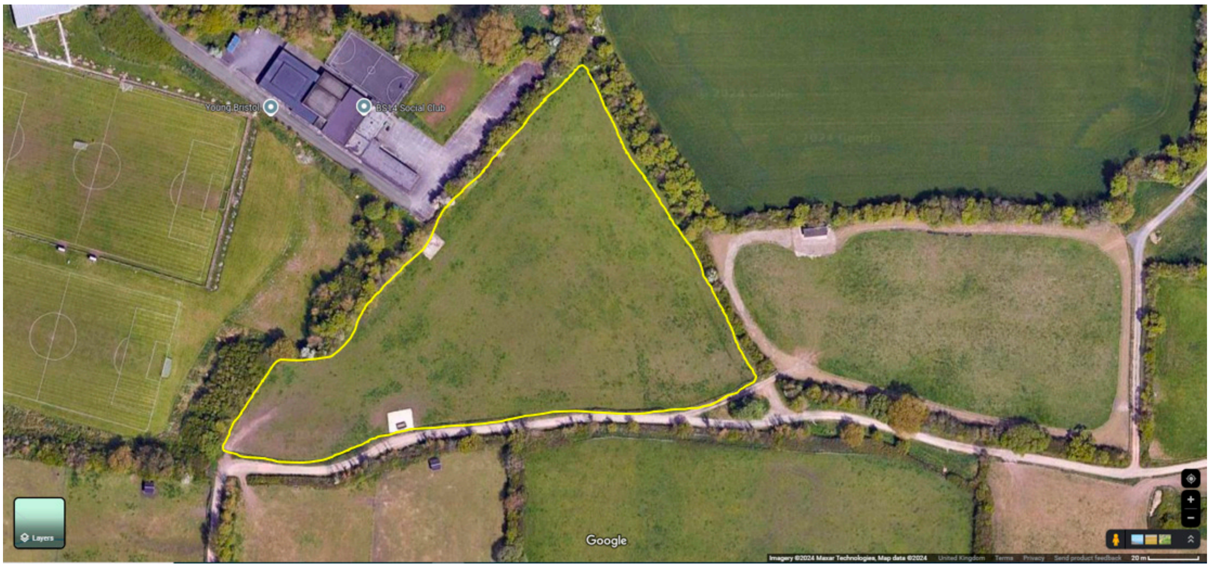

Figure S5 - Paddock E Area

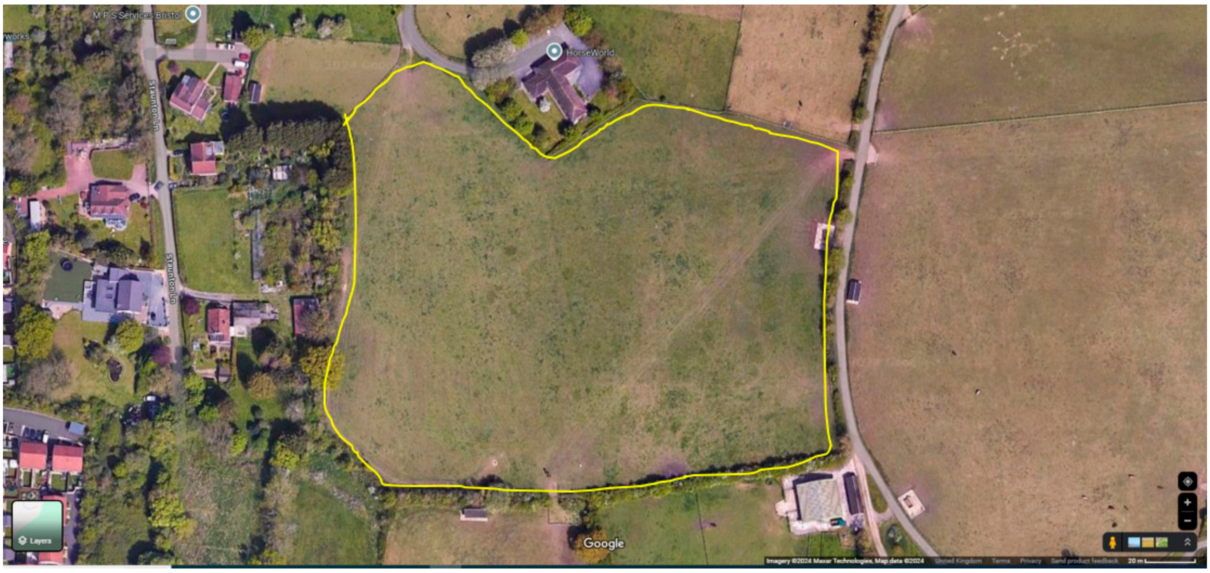

Figure S6 - Paddock F Area

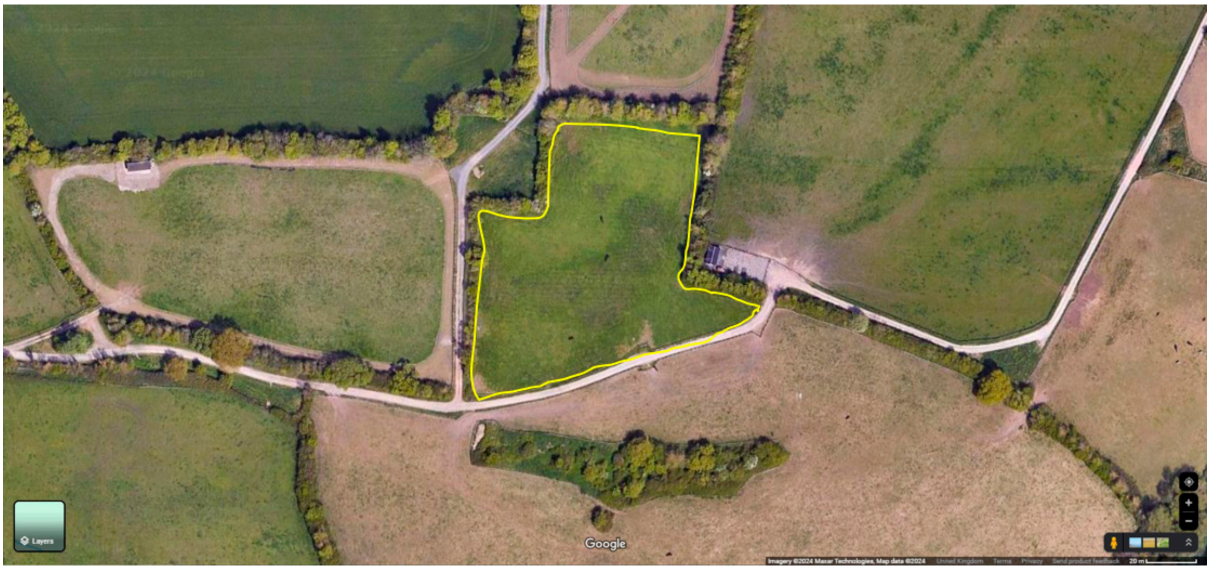

Figure S7 - Track A Layout

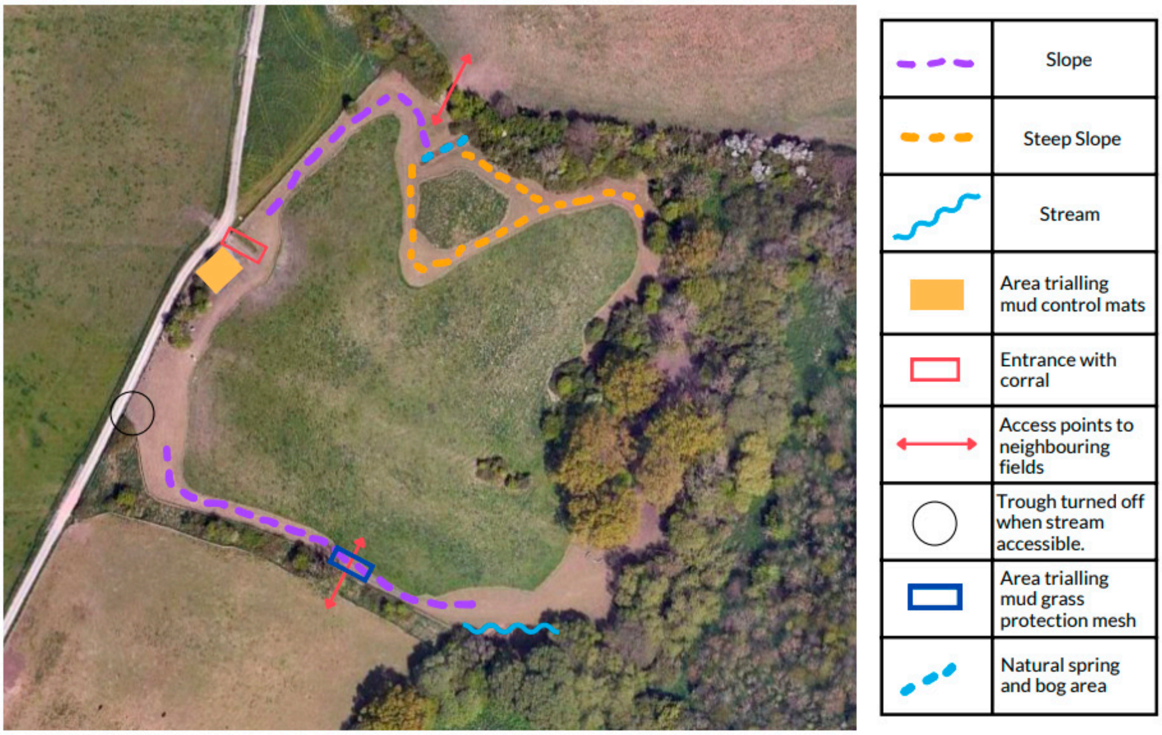

Figure S8 - Track B Layout

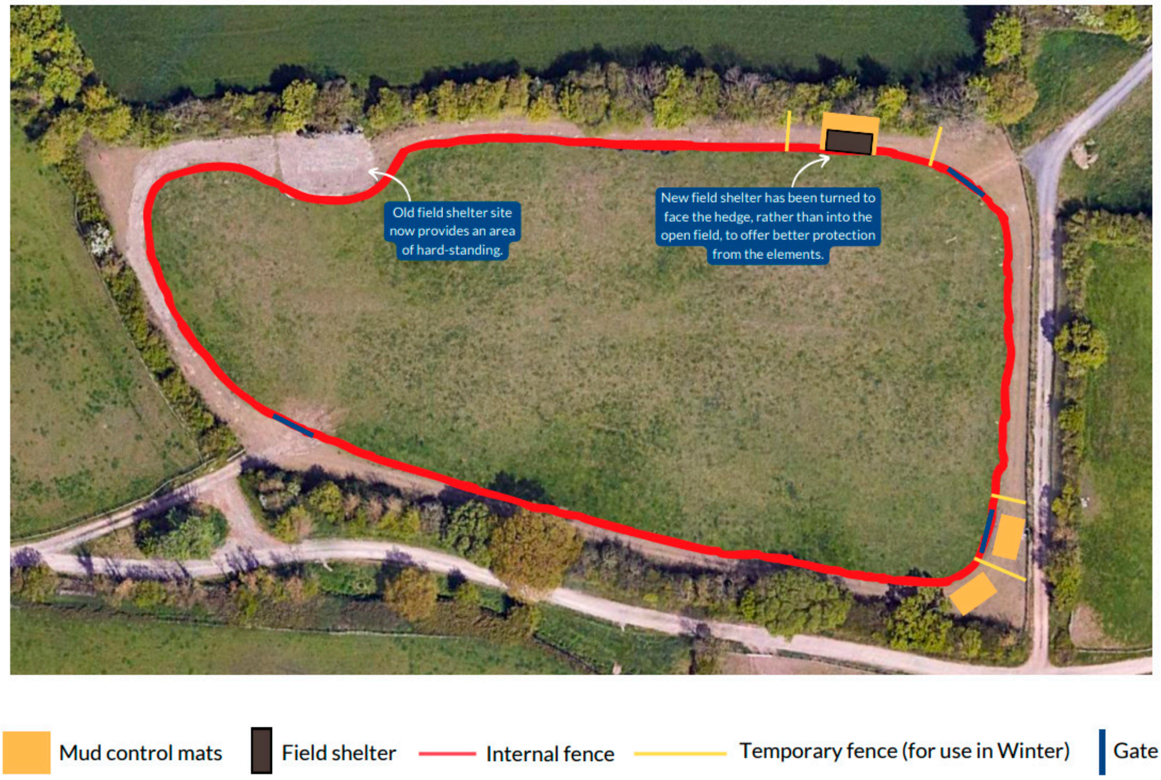

Figure S9 - Track C Layout

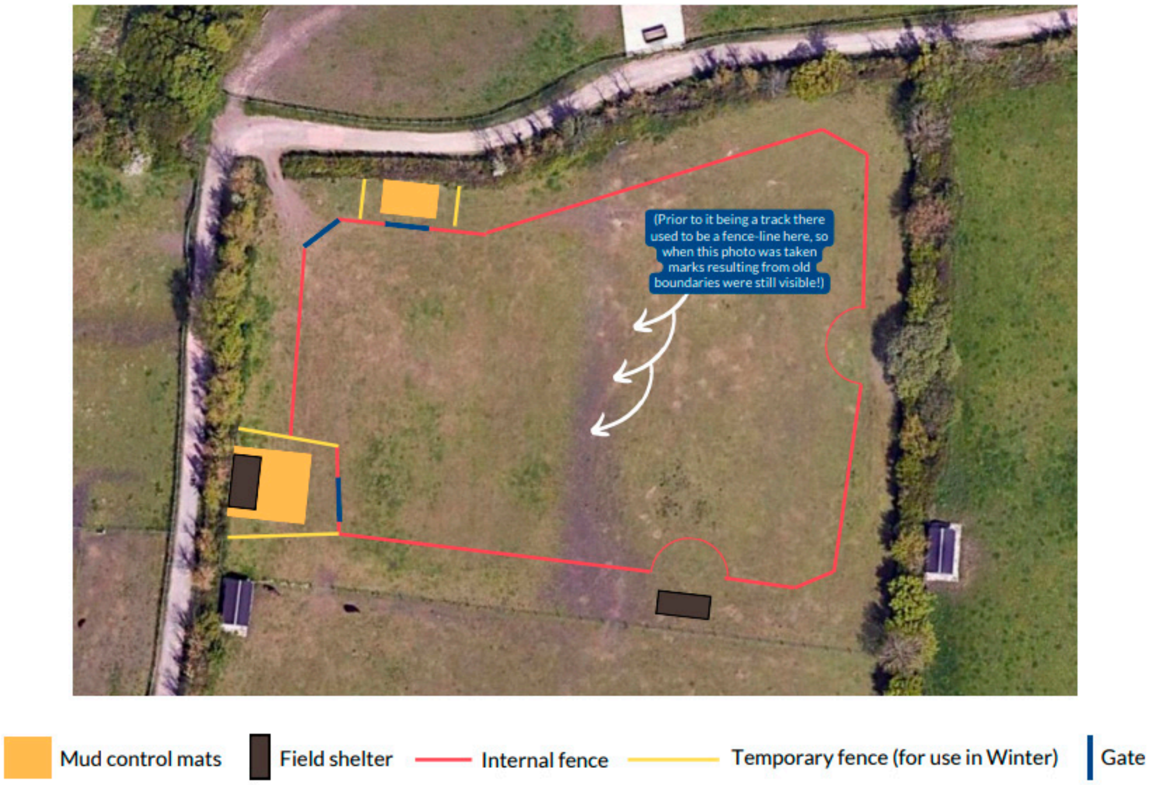

Supplement: Supplementary file 1 [file animals-15-00874-s001.zip › animals-3499236-supplementary.pdf]
